# A TRAF2 binding independent region of TNFR2 is responsibl for TRAF2 depletion and enhancement of cytotoxicity driven b TNFR1

**DOI:** 10.18632/oncotarget.1492

**Published:** 2013-11-29

**Authors:** Lucía Cabal-Hierro, Artime Noelia, Julián Iglesias, Prado Miguel A., Lore Ugarte-Gil, Casado Pedro, Belén Fernández-García, Bryant G. Darnay, Pedro S. Lazo

**Affiliations:** ^1^ Departamento de Bioquímica y Biología Molecular and Instituto Universitario de Oncología del Principado de Astur (IUOPA), Universidad de Oviedo, Oviedo, Spain.; ^2^ University of Texas. MD Anderson Cancer Center. Houston, Texas, USA.; ^3^ Present address: Abramson Cancer Center. Perelman School of Medicine, University of Pennsylvania Philadelphia, PA. US; ^4^ Present address: Department of Cell Biology. Harvard Medical School. Boston, MA. USA.; ^5^ Present address: Institute of Cancer, Barts Cancer Institute. Queen Mary University of London. London, UK

**Keywords:** TNF receptors, Death Receptors, TRAF2, Apoptosis, NF-kappaB

## Abstract

Tumor Necrosis Factor (TNF) interacts with two receptors known as TNFR1 and TNFR2. TNFR1 activation may result in either cell proliferation or cell death. TNFR2 activates Nuclear Factor-kappaB (NF-kB) and c-Jun N-terminal kinase (JNK) which lead to transcriptional activation of genes related to cell proliferation and survival. This depends on the binding of TNF Receptor Associated Factor 2 (TRAF2) to the receptor. TNFR2 also induces TRAF2 degradation. In this work we have investigated the structural features of TNFR2 responsible for inducing TRAF2 degradation and have studied the biological consequences of this activity. We show that when TNFR1 and TNFR2 are co-expressed, TRAF2 depletion leads to an enhanced TNFR1 cytotoxicity which correlates with the inhibition of NF-kB. NF-kB activation and TRAF2 degradation depend of different regions of the receptor since TNFR2 mutants at amino acids 343-349 fail to induce TRAF2 degradation and have lost their ability to enhance TNFR1-mediated cell death but are still able to activate NF-kB. Moreover, whereas NF-kB activation requires TRAF2 binding to the receptor, TRAF2 degradation appears independent of TRAF2 binding. Thus, TNFR2 mutants unable to bind TRAF2 are still able to induce its degradation and to enhance TNFR1-mediated cytotoxicity. To test further this receptor crosstalk we have developed a system stably expressing in cells carrying only endogenous TNFR1 the chimeric receptor RANK-TNFR2, formed by the extracellular region of RANK (Receptor activator of NF-kB) and the intracellular region of TNFR2.This has made possible to study independently the signals triggered by TNFR1 and TNFR2. In these cells TNFR1 is selectively activated by soluble TNF (sTNF) while RANK-TNFR2 is selectively activated by RANKL. Treatment of these cells with sTNF and RANKL leads to an enhanced cytotoxicity.

## INTRODUCTION

TNF interacts with two receptors known as TNFR1 and TNFR2. Soluble TNF (sTNF) only activates TNFR1, while membrane-bound TNF (mTNF) activates both receptors [1, 2]. Both TNFRs are highly similar in their extracellular regions which contain four cysteine-rich domains (CRD)[3]. However, TNFR1 and TNFR2 are quite different in their intracellular regions. TNFR1 contains a Death Domain (DD) responsible for triggering apoptosis whereas TNFR2 signals depend on the binding of several TRAF proteins and does not trigger apoptosis.

TNFR1 is a ubiquitous receptor the activity of which can result in either cell proliferation or cell death depending on the cell line and the microenvironmental conditions. Upon binding of TNF to TNFR1, different signalling complexes may be formed [4]. Complex I is a transmembrane complex leading to proliferative signals mainly mediated by NF-kB. The activation of TNFR1 also implies the internalization of the receptor and the formation of Complex II or DISC (Death Inducing Signalling Complex), implicated in the induction of apoptosis [5]. When caspases are inhibited however, Complex II is unable to trigger apoptosis, leading to the formation of a third complex named Necroptosome [6, 7] that is involved in the induction of necroptosis [8, 9].

TNFR2 is not so well characterized. The facts that TNFR2 expression is restricted to certain cell types [10] and that this receptor is activated only by mTNF [1] have contributed to this lack of information. After ligand binding, TNFR2 binds TRAF2 directly. Some other adaptor proteins such as TRAF1, TRAF3, Cellular Inhibitor of Apoptosis 1 (cIAP1) and cIAP2 bind to the receptor through TRAF2 [11, 12]. Thus, TRAF2 is a key mediator in receptor signalling [13]. TRAF2 binds to TNFR2 through two different sites in its intracellular region. The first site is comprised of amino acids 402-SKEE-405 and fits into the known TRAF binding site of the TNFR superfamily of receptors [12]. A second TRAF2 binding site has been recently identified by us [14] and is comprised of the last 15 amino acids at the C-terminal region of the receptor, being residues 425-KPL-427 directly responsible for this interaction. TNFR2 activation implies the induction of genes implicated in cell proliferation through NF-kB and N-terminal Jun Kinase (JNK) [15-17]. More recently it has been shown that TNFR2 and other receptors belonging to the TNFR superfamily induce TRAF2 degradation[18, 19]. We have shown that amino acids 343-349 are responsible for this action [14] although little is known about the molecular mechanisms implicated. TNFR2-induced TRAF2 depletion may lead to cell death because survival and proliferative signals triggered by both TNFR1 and TNFR2 depend on TRAF2. In this work we show that TRAF2 depletion leads to an enhanced cytotoxicity mediated by TNFR1 which correlates with the inhibition of NF-kB when both receptors are coexpressed and that TNFR2 mutants unable to induce TRAF2 degradation fail to enhance cytotoxicity. We also show that TRAF2 degradation does not rely on its interaction with the receptor. In an attempt to study independently the signals triggered by TNFR1 and TNFR2 as well as their croostalk we have developed a system stably expressing the chimeric receptor RANK-TNFR2 in cells carrying only endogenous TNFR1. RANK-TNFR2 triggers the same signals than TNFR2 but it is activated by RANKL. Thus, in these cells TNFR2 is selectively activated by RANKL while TNFR1 is selectively activated by sTNF. We show that, indeed, coactivation of TNFR1 and RANK-TNFR2 leads to an enhanced cytotoxicity provided that TNFR2 activation leads to TRAF2 depletion, rather than NF-kB activation.

## RESULTS

### TNFR2 affects the signals triggered by TNFR1

**Figure 1:**
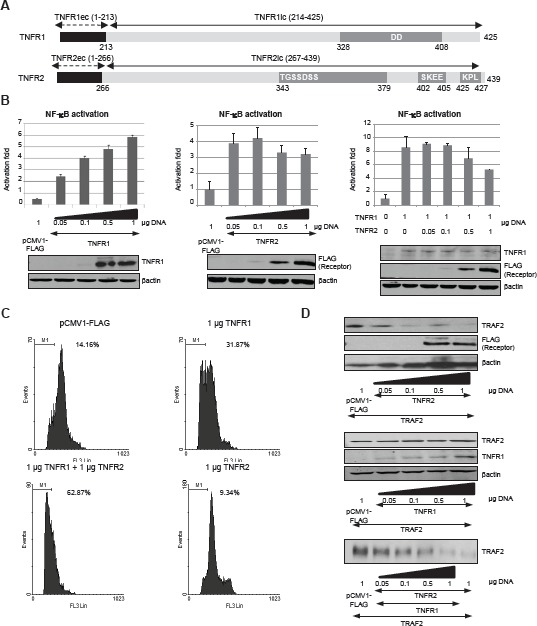
TNFR2 expression affects TNFR1 signalling A) Schematic representation of TNFR1 and TNFR2. Extracellular and transmembrane regions of each receptor are represented in black while the intracellular region is shown in light grey. In both receptors the main regions involved in their biological functions are also indicated: the DD in TNFR1 (amino acids 328-408), whereas in TNFR2 both the regions involved in TRAF2 depletion (amino acids 343-379) and TRAF2 binding sites (amino acids 402-405 and 425-427) are shown. B) NF-kB activation by TNF receptors. HEK 293 cells were transiently transfected with the indicated amounts of each expression vector encoding TNFR1 or TNFR2, as indicated, together with 0.5 µg of pNF-kB-luc and 0.5 µg of pRL-TK. Forty-eight hours later luciferase was determined in cell extracts as indicated in Materials and Methods. Expression of each receptor was also analyzed by western blot (anti-TNFR1 and anti-FLAG). C) Quantification of the sub G0/G1 population induced by TNF receptors. HEK293 cells were transfected with the indicated amounts of receptor-coding plasmids together with 0.2 µg of pGEGF-F. Forty-eight hours later, cells were harvested, stained with propidium iodide and analyzed by flow cytometry to quantify the sub G0/G1 population. The percentage of hypodiploid cells is indicated in each case. D) Analysis of the intracellular levels of TRAF2. HEK293 cells were transfected with the amount of the plasmids encoding the receptors indicated together with 0.5 µg of pTRAF2. Forty-eight hours later the intracellular levels of TRAF2 were determined by western blot.

TNFR1 and TNFR2 differ in their intracellular regions (Fig 1A). TNFR1 contains a Death Domain whereas TNFR2 contains no Death Domain. Instead, TNFR2 contains two TRAF2 binding sites [4]. Although TNFR1 contains no TRAF2 binding sites, TRAF2 appears responsible for triggering signals initiated by both receptors. Both TNFR1 and TNFR2 activate NF-kB [15, 20] Oligomerization of the receptors after ligand binding or by overexpression is the initial step in their function. We have studied TNFRs signalling by overexpressing them in the absence of the ligand.

Transient transfection of HEK293 cells with increasing amounts of either TNFR shows that the activation of NF-kB behaves differently depending on the receptor. Thus, when TNFR1 is expressed the activity of NF-kB increases as the amount of receptor increases (Fig 1B, left panel). On the other hand, when TNFR2 is expressed the activation of NF-kB shows a biphasic behaviour: at low receptor concentrations NF-kB activation increases with the amount of receptor but at high receptor concentrations NF-kB is inhibited (Fig 1B, middle panel). We have investigated the consequences of coactivation by overexpressing both receptors. As shown in Fig 1B (right panel) NF-kB is activated when both receptors are coexpressed. However, high TNFR2 concentrations lead to the inhibition of NF-kB, resembling what happens when TNFR2 alone is overexpressed. Interestingly enough, coexpression of TNFR1 and TNFR2 also lead to an enhanced cytotoxicity (Fig 1C). As expected, expression of TNFR1 caused apoptosis in HEK293 cells despite the fact that NF-kB was activated while expression of TNFR2 had no effect on cell viability. When TNFR1 and TNFR2 were coexpressed, the apoptotic effect of TNFR1 was enhanced (Fig 1C). Thus, TNFR2 activation has a dual consequence on TNFR1 signalling: a) it negatively affects the activation of NFkB and b) an enhanced cytotoxicity can be observed. These results point to a critical TNFR1-TNFR2 crosstalk in which TRAF2 appears as the critical mediator [21].When analyzing the intracellular levels of TRAF2 in cells expressing TNFR2 we observed that high receptor expression induces the depletion of the adaptor protein (Fig 1D, upper panel) which may explain why NF-kB is so poorly activated at these receptor concentrations (Fig 1B). As for TNFR1, this receptor is unable to induce TRAF2 degradation (Fig 1D, middle panel). Moreover, when both receptors are co-expressed together with TRAF2, TNFR2 also induces TRAF2 degradation, indicating that at high concentrations of both TNFR1 and TNFR2 the biological effects shown occur at low levels of TRAF2 (Fig 1D, lower panel).

### TRAF2 binding and TRAF2 degradation depend on different TNFR2 regions

**Figure 2:**
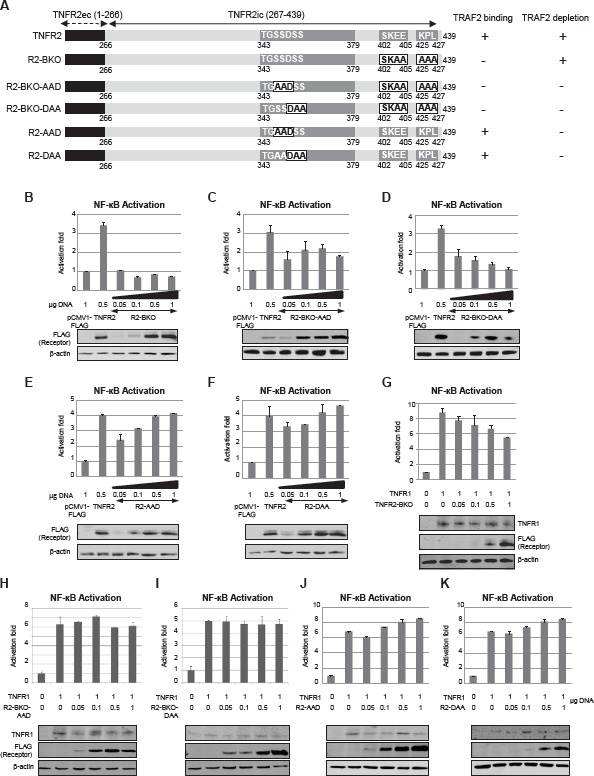
Effect of TNFR2-induced TRAF2 depletion on NF-kB activation by TNFR1 A) Schematic representation of TNFR2 and its mutants. Representation of the receptor is as in Figure 1A. Numbers indicate the amino acid position in the full-length human receptor. Letters in black indicate the mutated amino acids. Their activity for TRAF2 binding or TRAF2 degradation is indicated on the right. B-F) NF-kB activation caused by TNFR2 or its mutants. HEK293 cells were transiently transfected with the indicated amounts of each expression vector encoding TNFR2 or its mutants, together with 0.5 µg of pNFkB-luc and 0.5 µg of pRL-TK. Forty-eight hours later both luciferase activities were determined as described in Materials and Methods. Expression of the receptors was also determined by western blot. G-K) NF-kB activation caused by coexpression of TNFR1 and TNFR2 or its mutants. NF-kB activity was determined as above. Expression of TNFR1 and TNFR2 or its mutants was determined by western blot.

To study the role of TRAF2 depletion on NF-κB activation we generated several TNFR2 mutants in whichamino acids responsible for TRAF2 binding were mutated. We also generated several mutant receptors in which some of the amino acids comprising the TRAF2 degradation site were mutated (Fig 2A) [14]. After transfection of HEK293 cells with these mutant receptors the activation of NF-kB was determined. Mutation of amino acids 402-SKEE-405 to 402-SKAA-405 and amino acids 425-KPL-427 to 425-AAA-427 (receptor TNFR2-BKO) obliterated the ability of TNFR2 to induce NF-kB activation (Fig 2B) but it was still able to induce TRAF2 depletion. These amino acids comprise the two TRAF2 binding sites[12, 14]. Receptors in which aminoacids 343-349 were mutated were unable to induce TRAF2 degradation regardless of whether they were able to bind TRAF2. Thus, receptors unable to bind and to induce TRAF2 degradation (receptors TNFR2-BKO-AAD and TNFR2-BKO-DAA) showed a much lower capacity to activate NF-kB (Figs 2C and 2D) indicating that the interaction between the receptor and TRAF2 is essential for the activation of NF-kB. Moreover, when expressing TNFR2 mutant receptors able to bind TRAF2 but unable to induce its degradation (receptors TNFR2-AAD and TNFR2-DAA) the activation of NFkB increased as the amount of receptor was higher (Figs 2E and 2F). These receptors clearly differ from the wild type TNFR2 in which the activation of NF-κB shows a biphasic behaviour (Fig 1B), thus pointing to TRAF2 depletion as a regulatory action in TNFR2 signalling.

We next addressed the question of whether TNFR2-induced TRAF2 depletion affects the activation of NF-kB by TNFR1. For this purpose TNFR1 was coexpressed with increasing amounts of mutant receptors derived from TNFR2 and the activation of NF-kB was studied. The coexpression of TNFR1 and TNFR2-BKO (a receptor unable to bind TRAF2) resulted in a decrease of NF-kB activity as the amount of mutant receptor was increased (Fig 2G), similar to what happened when TNFR1 and wild type TNFR2 were coexpressed. However, when TNFR1 was coexpressed with a TNFR2 mutant unable to bind and to induce TRAF2 degradation, NF-kB activation was not affected by the mutant receptors (Figs 2H and 2I). Moreover, when TNFR1 was coexpressed with TNFR2 mutants in which the TRAF2 degradation site was mutated, NF-kB activity increased as the amounts of mutant receptors were increased (Figs 2J and 2K). These receptors (TNFR2-AAD and TNFR2-DAA) were able to bind TRAF2 and therefore to activate NF-kB. Taken together, these results show that TNFR2-induced TRAF2 depletion has a negative regulatory role on TNFR1 signalling and that the primary action of this regulatory role is the inhibition of NF-kB.

### TRAF2 depletion by TNFR2 is responsible for the enhancement of cytotoxicity driven by TNFR1

**Figure 3:**
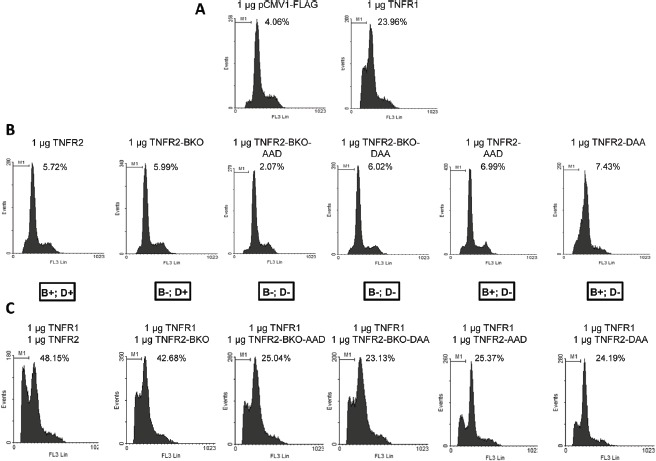
Effect of TNFR2-induced TRAF2 depletion on TNFR1 cytotoxicity HEK293 cells were transfected with: A) 1 µg of control plasmid or plasmid coding for TNFR1 plus 0.2 µg of pGEGF-F; B) 1 µg of the indicated receptor-coding plasmids plus 0.2 µg of pGEGF-F; C) 1 µg of the indicated receptor-coding plasmids and 1 µg of plasmid coding for TNFR1 plus 0.2 µg of pGEGF-F. TNFR2 and its mutants are named as in Figure 2 and the symbols B and D in the box under panel B refer to their ability to bind or to degrade TRAF2. Forty-eight hours later, cells were harvested, stained with propidium iodide and analyzed by flow cytometry to quantify the sub G0/G1 population. The percentage of apoptotic cells is indicated in each case.

As shown above, TNFR2 activation increases the cytotoxic effect of TNFR1 (Fig 1C). We have also shown that TRAF2 depletion by TNFR2 negatively affects NF-kB activation (Fig 1B). We therefore addressed the question of whether TRAF2 degradation could be linked to the increased cytotoxicity observed when TNFR1 and TNFR2 are coexpressed. For this purpose TNFR1, TNFR2 or mutant receptors derived from the latter were expressed in HEK293 cells and levels of cell death were determined. As expected, neither wild type TNFR2 nor mutant TNFR2s unable to bind TRAF2 or to induce TRAF2 degradation, were able to induce cell toxicity by themselves (Fig 3B). When these receptors were coexpressed with TNFR1 two different behaviours were distinguished. On one hand, when TNFR1 was coexpressed with TNFR2-BKO a enhanced cytotoxicity was observed which was comparable to that obtained by coexpression of TNFR1 and wild type TNFR2. TNFR2-BKO is a mutant receptor unable to bind TRAF2 or to activate NF-kB but that retains its ability to induce TRAF2 degradation. On the other hand, when TNFR1 was coexpressed with TNFR2 mutant receptors unable to degrade TRAF2, no enhancement of TNFR1 cytotoxicity was observed regardless of whether the receptor was able or not to bind TRAF2 (Fig 3C). All these results show once again that TRAF2 binding (and hence activation of NF-kB) and TRAF2 depletion are independent features of TNFR2 and also strongly suggest that TRAF2 depletion induced by TNFR2 is a key event in TNFR1-TNFR2 functional crosstalk. Thus, by lowering intracellular TRAF2 levels all NF-kB-dependent pathways are inhibited while TNFR1 induced cell death is enhanced.

### A chimeric receptor activated by RANKL but bearing the intracellular region of TNFR2 also enhances TNFR1 cytotoxicity

**Figure 4:**
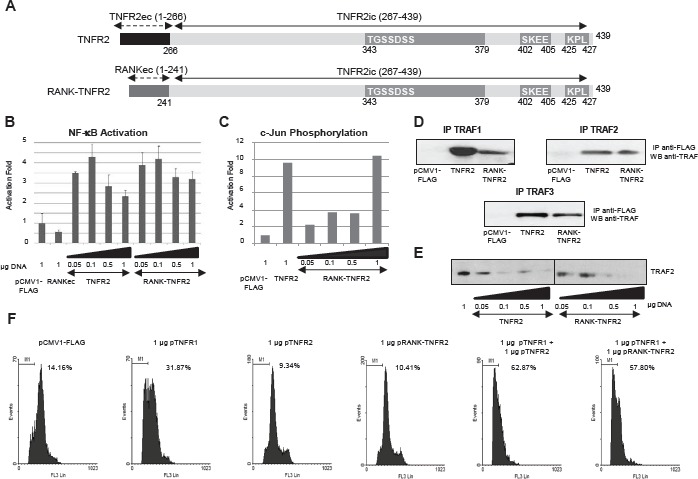
Functional analysis of the chimeric receptor RANK-TNFR2 A) Schematic representation of TNFR2 and RANK-TNFR2. The representation of the receptors is as in Fig. 1A. The chimeric receptor RANK-TNFR2 contains residues 1-241 corresponding to the extracellular and transmembrane domains of RANK and residues 267-439 corresponding to the intracellular region of TNFR2. B) NF-kB activation. HEK293 cells were transiently transfected with the indicated amounts of each expression vector encoding TNFR2 or RANK-TNFR2, together with 0.5 µg of pNF-kB-luc and 0.5 µg of pRL-TK. Forty-eight hours later luciferase was determined in all extracts. C) Analysis of c-Jun phosphorylation. HEK293 cells were transfected with the indicated amounts of TNFR2 or RANK-TNFR2 expression vectors and with 1 µg of pHA-JNK. After 48 hours, cells were harvested and JNK was immunoprecipitated followed by an in vitro c-Jun phosphorylation assay. Its levels were determined by western blot assay using anti-phospho-cJun. D) TRAFs interaction assay. HEK293 cells were transfected with 1 µg of each expression vector encoding TNFR2 or RANK-TNFR2 together with 0.5 µg of the plasmids encoding the adaptor proteins TRAF1, TRAF2 or TRAF3. Twenty-four hours later, the cells were harvested and the receptors immunoprecipitated. The presence of each adaptor protein in the immunoprecipitates was analyzed by western blot (anti-TRAF1, anti-TRAF2 or anti-TRAF3). E) Analysis of TRAF2 intracellular levels. HEK293 cells were transfected with 1 µg of the plasmids encoding the indicated receptors together with 0.5 µg of pTRAF2. Forty-eight hours later the intracellular levels of TRAF2 were determined by western blot analysis. F) Quantification of sub G0/G1 populations induced by TNFR2 or RANK-TNFR2. HEK293 cells were transfected with the indicated amounts of the plasmids encoding the receptors together with 0.2 µg of pGEGF-F. Forty-eight hours later cells were harvested, stained with propidium iodide and analyzed by flow cytometry to quantify the sub G0/G1 population. The percentage of apoptotic cells is indicated in each case.

The results reported up to now were obtained by transient overexpression of the receptors and their activation triggered by oligomerization in the absence of the ligand. We therefore addressed the question of whether in conditions in which the receptors are not overexpressed the TNFR1-TNFR2 crosstalk also occurs. Two different problems needed to be solved for this approach: a) although TNFR1 is ubiquitously expressed, TNFR2 expression is much more restricted in terms of cell type, b) TNFR2 is efficiently activated only by mTNF which also activates TNFR1; TNFR1 can be activated by soluble TNF as well. So, there are no specific ligands that independently activate TNFR1 and TNFR2. We solved this problem by generating a chimeric receptor RANK-TNFR2 in which the extracellular and transmembrane regions of Receptor Activator of NF-kB (RANK) were fused to the intracellular region of TNFR2 (Fig 4A). We first checked that this chimeric receptor is functionally equivalent to wild type TNFR2. Thus, by overexpressing this receptor in HEK-293 cells a biphasic activation of NF-kB was observed (Fig 4B). Also, RANK-TNFR2 induces c-Jun phosphorylation (Fig 4C), binds TRAF1, TRAF2 and TRAF3 (Fig 4D) and is also able to induce TRAF2 degradation (Fig 4E). Moreover, when the chimeric receptor was coexpressed with TNFR1 it also enhanced TNFR1-mediated cytotoxicity (Fig 4F).

In order to test the crosstalk between ligand-activated receptors, the chimeric receptor RANK-TNFR2 or its mutant RANK-TNFR2-Δ343 were stably expressed in the murine cell line L929. L929 cells do not express RANK and hence, NF-kB is not activated by RANKL (data not shown). Thus, the new L929 derived cell lines (L929-RANK-TNFR2) are the appropriate tools to study the signalling and the crosstalk of ligand-activated TNFR1 and TNFR2. In this system, TNFR1 is activated by sTNF and TNFR2 (RANK-TNFR2) is activated by RANKL. Ligand activated RANK-TNFR2 shows the same properties that were shown above for the transiently expressed receptor (data not shown).

**Figure 5:**
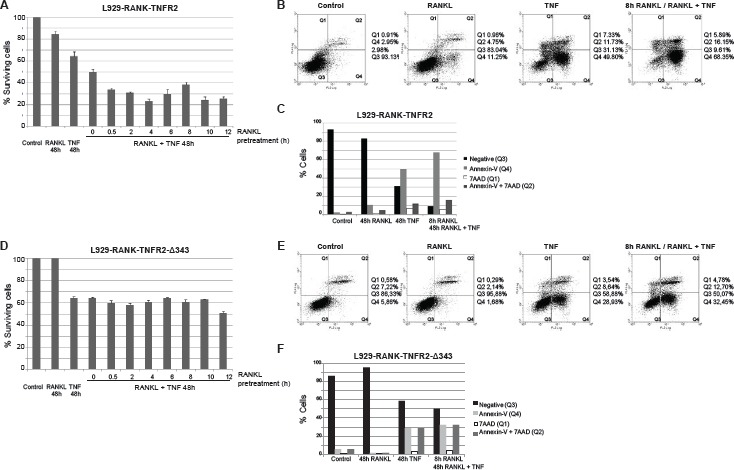
Enhancement of TNFR1-mediated cytotoxicity by activation of RANK-TNFR2 with RANKL Measurement of cell viability: L929 cells stably expressing the chimeric receptor RANK-TNFR2 (A) or RANK-TNFR2-Δ343 (D) were treated with soluble TNF to selectively activate TNFR1, pretreated with RANKL for the indicated times to selectively activate RANK-TNFR2 or with both cytokines. After 48 h cell viability was determined by staining with crystal violet and quantified as indicated in Materials and Methods. Data represent the quantification of such experiments carried out in triplicates. The error bars represent the error of the mean. Measurement of cell death markers. L929 cells stably expressing the chimeric receptor RANK-TNFR2 (B and C) or RANK-TNFR2-Δ343 (E and F)) were treated for 48 h with TNF, RANKL or both (in this case cells were pretreated with RANKL for 8h). Cells were harvested and apoptosis/ necrosis markers (AnnexinV or 7-AAD) were quantifed by fow cytometry as indicated in Materials and Methods. B and E panels show the flow cytometry analysis and the gating carried out. C and F panels show the quantification of the results.

We analyzed cell viability by measuring crystal violet staining when L929-RANK-TNFR2 cells were treated with RANKL, sTNF or both ligands simultaneously. Treatment of cells for 48 h with both sTNF and RANKL induced a significant increase in TNF cytotoxicity compared to that caused by TNF alone. When cells were pre-treated with RANKL for a period of as little as 0.5 h prior to the combined treatment, the increase in cytotoxicity was more pronounced. Thus, pre-treatment with RANKL for 4 h increased TNF cytotoxicity 48 h later from 40% to 80% (Fig 5A). We also analyzed the cell death markers annexin-V and 7 amino actinomycin D (7-AAD). As shown in Fig 5B and 5C, treatment with RANKL did not induce significant changes in either marker. Soluble TNF induced a very significant increase (from 2.95% to 49.8%) in annexin-V positive cells and also a significant increase (from 2.98% to 11.73%) in annexin-V/7-AAD positive cells. More importantly, co-treatment of cells with sTNF and RANKL further increased the percentage of annexin-V and annexin-V/7-AAD positive cells compared to the values obtained with sTNF alone (from 49.8% to 68.35% for annexin-V positive cells and from 11.73% to 16.15% for annexin-V/7-AAD positive cells, Fig 5B and 5C). To further confirm that the cytotoxicity observed after the combined treatment with RANKL and sTNF was due to TRAF2 depletion induced by TNFR2 we generated the L929-derived cell line named L929-RANK-TNFR2-Δ343. These cells stably express the chimeric receptor RANK-TNFR2-Δ343 in which the intracellular region has been deleted from aminoacid 343, therefore missing residues 343-349 responsible for TRAF2 degradation. Pretreatment of L929-RANK-TNFR2-Δ343 cells with RANKL did not induce any further increase in toxicity over the one caused by sTNF alone, as determined by crystal violet staining (Fig 5D). Similar results were observed when apoptosis markers were studied. Thus, treatment of L929-RANK-TNFR2-Δ343 cells with sTNF alone induced a significant increase in annexin-V positive cells (from 5.86% to 28.93%) though it was lower than in L929-RANK-TNFR2 cells (Fig 5E and 5F). Co-treatment of cells with sTNF and RANKL induced a very small increase in annexin-V or annexin-V/7-AAD positive cells compared to the values obtained with sTNF alone (Fig 5E and 5F). Altogether, these results confirm that ligand activation of TNFR1 triggers an apoptotic process that may be enhanced by ligand activation of TNFR2, provided that the signalling pathway leading to TRAF2 depletion prevails over the signalling pathways leading to NF-kB activation.

## DISCUSSION

Since TNFR1 and TNFR2 were identified [22], the biological functions of TNFR2 have remained elusive because of its restricted expression and also because it is only efficiently activated by membrane bound TNF that also binds and activates TNFR1. TNF binding to TNFR2 forces its trimerization which leads to its direct interaction with TRAF2 and also with TRAF1, TRAF3, cIAP1 and cIAP2 through their binding to TRAF2 [11, 23]. This results in the activation of NF-kB through both the cannonical and the non cannonical pathways [15, 16, 24] and JNK[25] which lead to transcriptional activation of genes related to cell proliferation and survival. Thus, TRAF2 acts as a key mediator in TNFR2 signalling [11].The translocation of this Signalling Complex to the cytoplasm depends on cIAP1 and cIAP2. Several intermediates of the interaction TRAF2-cIAPs have been identified [26] [27]. Also, MEKK1 undergoes an autophosphorylation that activates it, leading to the late phosphorylation and activation of JNK [17].

TNFR2 activation induces changes in the subcellular localization of TRAF2 [28, 29]. Upon TNFR2 stimulation TRAF2 translocates to a new compartment[16, 19, 30, 31] where the adaptor protein is K48-linked, ubiquitinated and finally degraded by the proteasome [18, 29, 32, 33] although it has been also proposed that TRAF2 degradation takes place in the lysosome [34], being cIAP1 identified in both cases as the E3 ligase responsible for TRAF2 degradation. We have shown that NF-kB is activated with different efficiencies as increasing amounts of TNFR2 are expressed and that the extent of activation correlates with changes in TRAF2 concentration [14]. Amino acids 343-349 of the receptor appear to be responsible for the induction of TRAF2 degradation, which negatively regulates TNFR2-induced NF-kB activation. This region is constituted of 6 serine residues of which 4 show a high score for being phosphorylation sites, thus pointing to a phosphorylation of the receptor as the origin of TRAF2 depletion. Mutations of these serine residues abolish the ability of TNFR2 to degrade TRAF2 [14]. Interestingly, whereas signal triggering requires TRAF2 binding to the receptor, signal termination (TRAF2 depletion) appears independent of TRAF2 binding. Thus, a TNFR2 receptor unable to bind TRAF2 is still able to induce its degradation. TNF can induce another TRAF2 ubiquitination pattern, implicating K63-linked polyubiquitin chains, a modification not related to degradation but to JNK activation [32]. In this regard TRAF2 interaction with ubiquitin proteases such as USP31 (Ubiquitin Specific Protease) or CYLD (Cylindromatosis) which show specificity for K63-linked polyubiquitin chains is interesting [35, 36]. It is therefore conceivable that TRAF2 is initially modified through K63-linked polyubiquitin chains, converted into an active signal transducer and then, through K48-linked polyubiquitin chains, tagged for degradation, thus terminating TRAF2-dependent signals.

TNFR2 does not present a DD domain on its intracellular region and it is therefore unexpected of inducing cell death by itself. However, some studies have pointed to the fact that TNFR2 is able to induce cytotoxicity [37, 38]. Several researchers have pointed to the fact that TNFR2 could potentiate TNFR1 cytotoxicity[14, 39, 40]. In one case it has been shown that it does so by the inducing TNF production[41]. Here we show that TRAF2 depletion by TNFR2 is a negative feedback for NF-kB activation which has an important effect on TNFR1 signalling, resulting in the increased cytotoxicity induced by this receptor. This crosstalk appears to not only affect TNFR1 since the activation of apoptosis by FASL (Fas ligand) has also been reported to be affected by TNFR2 [42]. Thus, the ability of TNFR2 to induce TRAF2 degradation and therefore its depletion results in the inhibition of the antiapoptotic pathways and in the enhancement of the cytotoxicity mediated by TNFR1 when both receptors are coexpressed and activated. For an in depth characterization of the crosstalk between both TNF receptors we have studied the implication of different TNFR2 regions on TNFR1 signalling. It was known that TNFR2 signalling relies on TRAF2 binding to the region 402-SKEE-405 of the receptor [12]. We have shown that TNFR2 presents a second TRAF2 binding site located at the C-terminal region of the receptor. A third site, which is TRAF2 binding independent, is responsible for TNFR2 ability to induce TRAF2 degradation [14]. Here we show that this TNFR2 region involved in TRAF2 degradation is responsible for the increase in TNFR1 cytotoxicity when both receptors are activated. Evidence that this crosstalk relies on TRAF2 depletion comes from our results in which coexpression of TNFR1 and TNFR2 mutants unable to degrade TRAF2 does not affect TNFR1 cytotoxicity (Fig 2 and Fig 3). TRAF2 (as well as the anti-apoptotic proteins cIAP1 and cIAP2) interaction with TNFR1 inhibits the ability of this receptor to induce cell death [39, 40]. When TNFR1 and TNFR2 are coactivated, TNFR2 induces TRAF2 degradation thus affecting the formation of TNFR1 signalling complex, preventing the binding of cIAP1 and cIAP2 to TNFR1 which **finally** enhance its apoptotic potential. In this regard it is interesting that TRAF1 together with TRAF2 have been shown to suppress TNFR1 activation of apoptosis [43]. Thus, the balance between the apoptotic and antiapoptotic signals triggered by TNF determines whether the final outcome will be either cell death or cell survival. In other words, by activating the same set of receptors biological effects as diverse as proliferation or cell death can be obtained depending on the quantitative balance between the pro- and anti-apoptotic signals. Thus, it is particularly interesting to study the regulatory aspects of TRAF2 degradation. The coexpression of TNFR1 with TNFR2 mutant receptors unable to either bind or degrade TRAF2 reinforces the role of the adaptor protein as a key element in receptor crosstalk. Our results show that NF-kB activation only occurs when TNFR2 is unable to induce TRAF2 depletion (Fig 2). Conversely, the cytotoxic activity of TNFR1 increases when the receptor is expressed together with a TNFR2 receptor that induces TRAF2 degradation.

The absence of an exclusive ligand for TNFR2 is the major limiting factor for the study of this receptor. In this study we describe the generation of a functional system to study TNFR2 itself and also to study TNFR1/ TNFR2 crosstalk. This meant the stable expression of a chimeric RANK-TNFR2 receptor in a cellular context free of RANK. Thus, cell treatment with RANKL activates TNFR2 whereas sTNF activates TNFR1. We show that cells treated with both RANKL and sTNF are killed more efficiently than those treated with sTNF alone (Fig 5), indicating again that the activation of TNFR2 causing TRAF2 depletion is a biological mechanism to enhance TNFR1 cytotoxicity. In this work we show that aminoacids 343-349 of TNFR2 have a principal role on the signalling pathways triggered by the receptor. Although it is not involved in TRAF2 binding it clearly regulates its intracellular levels, thus modulating NF-kB activation and, subsequently, TNFR1 cytotoxicity.

In addition to NF-kB, the JNK pathway is almost invariably activated by TNF receptors. Blocking of the NF-kB dependent pathway leads to sustained JNK activation and apoptosis and, conversely, the blocking of TNF-induced JNK pathway promotes cell survival [44-46]. JNK induces cell death through mitochondrial events [47, 48]. In most cell types JNK activation by TNF does not imply cell death because TNF induces a transient activation of JNK [25]. Thus, it is likely that TNFR2-TRAF2 interaction leads to a transient activation of JNK that implies the activation of proliferative pathways whereas a further and sustained activation of the kinase could lead to cell death in the context of NF-kB inhibition. This agrees with the fact that JNK is efficiently activated in the conditions reported in this work (results not shown). Recent data point to the interaction of the adaptor protein AIP1 (ASK1-interacting protein 1, involved in JNK activation) with TNFR2 suggesting a TRAF2-independent JNK activation by TNFR2 [49]. AIP1 also interacts with TNFR1, being involved in JNK activation and apoptosis signalling [50].

## MATERIALS AND METHODS

### Cell lines and transfection methodology

Human embryonic kidney 293 (HEK 293) and murine fibroblastic L929 cell lines were obtained from the American Type Culture Collection (Rockville, MD).GP2 cell line was purchased from Clontech. HEK293 and GP2 cells were cultured in minimal essential medium supplemented with 10% fetal bovine serum, 1% glutamine, 0.13% bicarbonate and antibiotics (100 U/ ml Penicillin, 50 mg/ml Streptomycin). L929 cells were cultured in Roswell Park Memorial Institute medium supplemented with 10% fetal bovine serum and antibiotics (100 U/ml Penicillin, 50 mg/ml Streptomycin). For transient transfections, HEK 293 cells (2 x 10^5^ cells/well on 6-well plates) were seeded and transfected with 4.25 µg polyethylenimine (PEI) pH 7.5 with the indicated plasmids in each case in 200 µl of DMEM sumplemented with 1% of non-esential aminoacids following manufacturer's instructions. For stable transfection of the L929 cells, the packaging cell line GP2 was used. GP2 cells (0.6 x 10^5^ cells/well on 6 well-plates) were seeded and transfected with 3 µg of pBABE-puro-RANK-TNFR2 and 1 µg of pVSVg using Fugene and following manufacturer's instructions in 1ml of αMEM medium. Twenty-four hours later, 2 ml of αMEM medium was added to each well and 2 additional ml more 24 hours later. Seventy-two hours after transfection the medium of GP2 cells containing the retroviral particles was collected to infect L929 cells together with 10 µg/ml of Polibren. After 72 hours, the positive clones were selected by adding 4 µg/ml of puromycin to the medium.

### Plasmids and antibodies

Expression plasmid encoding human Myc-tagged TRAF2 as well as the plasmids encoding the adaptor proteins TRAF1 and TRAF3 have been previously described [51]. The NF-κB reporter construct pNF-κB-luc encodes luciferase from firefly (*Photinus pyralis*) under the control of a promoter with several κB elements. It was a generous gift from Dr. David S. Ucker, from the University of Illinois at Chicago. The pRL-TK plasmid (Promega) encodes luciferase from *Renilla reniformis* under the control of the Thyminide Kinase promoter from HSV-TK herpes virus. The HA-JNK encodes JNK protein tagged with the HA antigen. It was a generous gift from Dr. Pilar de la Peña, from the University of Oviedo. pEGFP-F expresses a farnesylated version of the green fluorescent protein GFP and was also a generous gift from Dr. David S. Ucker.

Expression plasmids encoding human FLAG-tagged TNFR2 (pCMV1-FLAG-TNFR2) and human TNFR1 (pCDNA3-TNFR1) were a gift from B.B. Aggarwal (MD Anderson Cancer Center, Houston, Texas, USA). Unless otherwise indicated, TNFR2 constructs used in this work were generated by PCR using standard methods and the primers indicated in Table SI. To generate point mutations by PCR mutagenesis, the Quick Change Site-Directed Mutagenesis Kit of Strategene was used together with the primers indicated in Table SI. The sequences of all plasmids generated in this work were verified by automated DNA sequencing

Primary antibodies against TRAF1 (G-20), TRAF2 (C-20), TRAF3 (H-20) and TNFR1 (H-5) were obtained from Santa Cruz Biotechnology, Inc. (Santa Cruz, CA, USA). Anti-FLAG (F3165) and anti-κ-actin (A5441) from Sigma. Anti-HA antibody was purchased from Roche and anti-c-Jun-phospho-Ser73 antibody was obtained from Cell Signalling. Secondary antibodies anti-rabbit IgG and anti-mouse IgG tagged with fluorophores were purchased from LICOR-Biosciences.

### Western blotting

Proteins were separated by SDS-PAGE, electroblotted onto PVDF membranes (Immobilone-FL, Millipore), blocked for 1 hour in 5% non-fat milk and incubated with the indicated primary antibodies (at 1:5,000 dilution in TBS-0.1% Tween) and the appropriate secondary antibody (at 1:15,000 dilution in 5% non-fat milk in TBS-0.1% Tween). Membranes were scanned with the Odyssey® Infrared Imaging System (LI-COR biosciences).

### Transcriptional activity of NF-kB

NF-kB activity was determined analyzing the expression of luciferase. HEK293 cells were transfected with 0.2 µg of pNF-kB-luc, 0.05 µg of pRL-TK and with the amounts of the plasmids of interest indicated in each case. The activities of both luciferases were determined with the Dual-LuciferaseTM Reporter Assay System kit (Promega) following manufacturer's instructions. Basal activity was considered the one obtained in cells transfected with the pCMV1-FLAG vector alone. In all cases the data are represented as activation fold over the control condition, once corrected the value of firefly luciferase activity with the value of Renilla luciferase activity.

### Quantification of the hypodiploid cell population

HEK293 cells were transfected with the plasmids of interest together with 0.2 ug of pEGFP-F. After 36 hours the cells were collected (including any floating cells in the culture medium), washed twice with PBS and permeabilized with 1 ml of 70% Ethanol at -20°C added drop by drop while vortexed softly. Cells were incubated overnight at -20°C. Next, the samples were washed twice in PBS and resuspended in 400 µl of 5 µg/ ml PI, 100 µg/ml RNAse in PBS for 15 min in the dark at room temperature. Finally, the cell cycle was analysed by flow cytometry (Cytomics FC500, Beckman Coulter) to quantify variations in the sub G0/G1 population of the transfected cells.

### c-Jun phosphorylation

HEK293 cells were transfected with 1 µg of pHA-JNK and with the plasmids of interest indicated in each case. After 36 hours, cells were harvested and resuspended in 200 µl of lysis buffer (20 mM Tris-HCl pH 7.5, 150 mM NaCl, 1 mM EDTA, 1% Triton X-100, 2.5 mM sodium pyrophosphate, 1 mM β-glicerophosphate, 1 mM sodium orthovanadate, 1µg/ml leupeptin, 1mM PMSF) on ice for 5 minutes and centrifugated for 15 minutes at 13,000 rpm at 4 °C. Equivalent amounts of proteins of each sample were incubated with 1 µg of anti-HA for 4 hours in rotation at 4 °C. Next, each sample was incubated with 20 µl of G-protein linked to a Sepharose matrix overnight in rotation at 4 °C. The samples were then washed twice with 500 µl of lysis buffer and also twice with 500 µl of kinase buffer (25 mM Tris-HCl pH 7.5, 5 mM β-glicerophosphate, 2 mM DTT, 0.1 mM sodium orthovanadate, 10 mM MgCl_2_) for 2 minutes at 5,000 rpm at 4 °C. The complexes were incubated in 40 µl of kinase buffer supplemented with 200 µM ATP and 1 µg of GST-c-Jun (Cell Signalling). The reaction was incubated at 30 °C for 30 minutes and then stopped with 40 µl of Laemmli buffer (62.5 mM pH 6.8 Tris-HCl, 2% SDS, 10% glycerol, 50 mM DTT, 2.5% β-mercaptoethanol and 0.01% bromophenol blue). The samples were analyzed by Western-Blot using the primary antibody anti-cJun-phospho-Ser73.

### Co-immunoprecipitation assay

HEK 293 cells were cotransfected with 1μg of the plasmids indicated in each case together with 0.3 μg of the plasmids encoding the TRAF protein of interest. Twenty-four hours after transfection, cells were harvested and lysed in 200 μl of Lysis buffer (20 mM pH 8.0 Tris-HCl, 150 mM NaCl, 1 mM DTT, 2 mM EDTA, 1% Triton X-100, 1 μg/μl leupeptin, 1 μg/μl aprotinin, 2 mM sodium orthovanadate). Two 20 μl fractions were collected as inputs for the TRAF protein and for the receptor as expression controls. The remaining fraction was incubated with 1μg of anti-FLAG antibody at 4 °C in rotation for one hour and then with 20 μl of protein G-Sepharose beads in rotation at 4 °C overnight. After three washes with lysis buffer and an additional one with low salt buffer (20 mM pH 8.0 Tris-HCl, 25 mM NaCl, 1 mM DTT, 1 μg/μl leupeptin, 1 μg/μl aprotinine, 2 mM sodium orthovanadate), the immunoprecipitate was resuspended in SDS-sample buffer and subjected, together with inputs, to SDS-PAGE and Western blot with anti-TRAF1, TRAF2 or TRAF3 and anti-FLAG (anti-receptor).

### Analysis of cellular viability

L929 cells were seeded in 12-well plates (1x10 ^5^ cells/plate) and treated 24 hours later with 5 ng/ml of RANKL or TNF or with both cytokines at the times indicated. To study the cell viability under each treatment, cells were washed once with PBS and fixed with 500 μl of a buffer containing 30% ethanol and 20% acetic acid during 15 minutes. Next, cells were stained with a 0.4% crystal violet solution in 20% ethanol during 15 minutes and washed once with H_2_O. After drying the plates overnight, they were destained during 20 minutes with 500 μl of a buffer containing 40% ethanol and 10% acetic acid. To quantify the amount of dye retained in each condition, the absorbance of each sample at 580 nm was determined.

### Analysis of cell death induction

To study the induction of a cell death process, the percentage of positive cells for AnnexinV and 7-AAD was determined using the AnnexinV PE Apoptosis Detection Kit I from BD Pharmingen following the manufacturer's instructions. Briefly, L929 cells were seeded in 12-well plates (1x10 ^5^ cells/plate) and 24 hours later treated with 5 ng/ml of RANKL or TNF or with both cytokines at the times indicated. Cells were harvested and washed twice with PBS. The percentage of positive cells for Anexin-V and 7-AAD was then determined by flow cytometry on a Cytomics FC500 (Beckman Coulter) cytometer.

## Supplemental and Table


