# Supplementary material for: A TRAF2 binding independent region of TNFR2 is responsibl for TRAF2 depletion and enhancement of cytotoxicity driven b TNFR1
Source: Oncotarget. 2013 Nov 29;5(1):224–36. doi: 10.18632/oncotarget.1492 (PMC3960203; doi:10.18632/oncotarget.1492)
Supplement: Supplementary file 1 [file oncotarget-05-0224-s001.doc]

**Table SI**

Oligonucleotides used to generate TNFR2 mutants and deletions used in this work

| **Receptor** | **Sequence 5´-3** | **Primer Sense** | **Comments** | **Template** |
| --- | --- | --- | --- | --- |
| **pCMV1-FLAG-TNFR2-BKO** | GAGCACCGAAGAGGCGGCCGCGCCCCTTGG | Sense | Point mutations underlined | pCMV1-FLAG-TNFR2-SKAA |
|  | CCAAGGGGCGCGGCCGCCTCTTCGGTGCTC | Antisense |  |  |
| **pCMV1-FLAG-TNFR2-BKO-AAD** | CAGCACCGGGGCCGCAGATTCTTCCC | Sense | Point mutations underlined | pCMV1-FLAG-TNFR2-BKO |
|  | GGGCAGAATCTGCGGCCCCGGTGCTG | Antisense |  |  |
| **pCMV1-FLAG-TNFR2-BKO-DAA** | GAGCTCAGATGCTGCCCCTGGTGGC | Sense | Point mutations underlined | pCMV1-FLAG-TNFR2-BKO |
|  | GCCACCAGGGGCAGCATCTGAGCTC | Antisense |  |  |
| **pCMV1-FLAG-TNFR2-AAD** | CAGCACCGGGGCCGCAGATTCTTCCC | Sense | Point mutations underlined | pCMV1-FLAG-TNFR2 |
|  | GGGCAGAATCTGCGGCCCCGGTGCTG | Antisense |  |  |
| **pCMV1-FLAG-TNFR2-DAA** | GAGCTCAGATGCTGCCCCTGGTGGC | Sense | Point mutations underlined | pCMV1-FLAG-TNFR2 |
|  | GCCACCAGGGGCAGCATCTGAGCTC | Antisense |  |  |
| **pCMV1-FLAG-RANK-TNFR2** | AGCTGAATTCCTGCAGAGAGAAGCCAAGGTGCC | Sense | *EcoRI* site underlined | From pCMV1-FLAG-TNFR2 into pCMV1-FLAG-RANKec |
|  | AGCTGTCGACAGGCTGGTGGGCACTGGAGTG | Antisense | *SalI* site underlined |  |
| **pBABE-puro-RANK-TNFR2** | agctTACGTACCCGCTGTTCGCGCTG | Sense | *SnaBI* site underlined | From pCMV1-FLAG-RANK-TNFR2 into pBABE-puro |
|  | agctTACGTACAGGGGCTTCTCTT | Antisense | *SnaBI* site underlined |  |
